# Opioid Prescribing Patterns For Adolescents Undergoing Posterior Spinal Fusion for Adolescent Idiopathic Scoliosis: A Multi-Site Retrospective Study

**DOI:** 10.7759/cureus.106196

**Published:** 2026-03-31

**Authors:** De-An Zhang, Lloyd M Halpern, Sundeep S Tumber

**Affiliations:** 1 Pediatric Anesthesia, Shriners Children's Southern California, Pasadena, USA; 2 Pediatric Anesthesia, Shriners Children's Spokane, Spokane, USA; 3 Pediatric Anesthesia, Shriners Children's Northern California, Sacramento, USA

**Keywords:** adolescent idiopathic scoliosis, gabapentin, multimodal analgesia, muscle relaxant, opioid prescribing, posterior spinal fusion

## Abstract

Introduction: As the opioid crisis has evolved from prescription drug abuse to illicit fentanyl use, research has identified leftover surgical medications as still being a significant source of misuse. While the CDC provides quantitative opioid prescribing benchmarks for adults, discrete numerical targets for the pediatric and adolescent populations remain poorly defined. This study evaluates longitudinal trends in opioid prescribing patterns for adolescents undergoing posterior spinal fusion (PSF) for adolescent idiopathic scoliosis (AIS) across a large, multi-site healthcare system.

Methods: We conducted a retrospective review of electronic medical records from eight pediatric hospitals for patients with AIS undergoing primary PSF from January 1, 2018, to August 1, 2024. Opioid-naïve patients with a valid discharge opioid prescription were included (n=1,034). Primary outcomes included total morphine milligram equivalents (MME) per prescription, maximum morphine milligram equivalents daily (MMED), the number of doses prescribed, and the 30-day refill rate. Statistical trends over time were analyzed using Kruskal-Wallis and Cochran-Armitage tests.

Results: The cohort was 76% female with a mean age of 15.4 ± 2.07 years. Oxycodone accounted for 94% of initial prescriptions. From 2019 to 2024, there was a statistically significant decrease in the mean total MME prescribed per patient (321.4 mg to 200.7 mg, p < 0.001). This decline was primarily driven by a reduction in the number of doses (35.2 to 24.0, p < 0.001) rather than the MME per dose. The proportion of patients receiving <50 MMED increased significantly from 68% to 82% (p = 0.003), while only 5% of the total cohort exceeded the 90 MMED adult threshold. The overall refill rate was 14% and remained stable throughout the study period despite the reduction in initial prescription volumes. Significant variability in prescribing quantities was observed between the eight study sites (p < 0.05).

Conclusion: Total opioid MME prescribing for adolescent PSF has significantly decreased, mainly due to reduction in total quantity over the duration of the study without a concomitant increase in refills. However, the inter-site variability between sites underscores the need for standardized, evidence-based, and procedure-specific quantitative guidelines for PSF to optimize postoperative pain management while minimizing unused opioids.

## Introduction

Over the past two decades, the characteristics of the opioid crisis have shifted markedly. What began as a rise in prescription opioid availability and misuse (driven by increased prescribing in the late 1990s and early 2000s) transitioned to illicit opioid markets dominated first by heroin and then by synthetic opioids, principally fentanyl [1]. Despite declines in national opioid dispensing since the late 2010s, opioid-related mortality rates have continued to increase. Many people who develop opioid use disorder first encountered opioids through legitimate prescriptions for acute or chronic pain [2]. Leftover medications from medical care are a well-documented source of misuse and diversion [3,4]. Prescription opioids still fuel misuse through ongoing prescribing and the large reservoir of unused pills that can be diverted. 

In 2016, the Centers for Disease Control and Prevention (CDC) released opioid dosing guidance for adults greater than or equal to 18 years of age. These initial guidelines advised avoiding opioid prescriptions greater than 90 morphine equivalents daily (MMED) [5]. The CDC updated adult opioid prescribing guidelines in November of 2022, extending the focus of the 2016 prescribing guidelines from chronic pain to acute, subacute, and chronic pain [6]. Recommendations for opioid-naïve patients include using the lowest starting dose possible, approximately 5-10 morphine milligram equivalents (MME) per dose or 20-30 MMED [6]. The firm recommendation to prescribe less than 90 MMED was removed, but guidelines continue to recommend caution above 50 MMED, noting decreased improvement in pain and function with increased risk at this level [6].

In pediatrics, opioid prescribing guidelines have been released by the American Academy of Pediatrics for acute pain management in the outpatient setting [7] and for pain following surgery by the American Pediatric Surgical Association [8]. Neither source establishes a discrete quantitative threshold comparable to the CDC guidelines. In the absence of widely accepted adolescent-specific opioid prescribing guidelines, clinicians often adapt adult frameworks to younger populations while applying clinical considerations, including weight-based dosing, developmental pharmacology, and caregiver involvement.

Given advances in clinician, patient, and patient’s guardian knowledge about the opioid crisis and advancements in multi-modal analgesia protocols, we reviewed our healthcare system’s opioid prescribing patterns from 1/1/2018 to 8/1/2024 for adolescent idiopathic scoliosis (AIS) patients undergoing posterior spinal fusion (PSF) with instrumentation. We hypothesized that we would observe a continuing decline in the amount of opioids prescribed. 

## Materials and methods

This study was reviewed and approved by the WCG Institutional Review Board (WCG-IRB; an AAHRPP-accredited independent IRB that serves as IRB of record for multisite studies). In accordance with U.S. federal regulations (45 CFR 46.116), WCG-IRB granted a waiver of informed consent given that the study posed minimal risk and involved retrospective review of de-identified medical records. Data access and use were conducted under institutional data use agreements with the contributing pediatric health system(s).

In 2018, our nationwide pediatric health system began a phased rollout of electronic prescribing. Use of electronic prescribing was the norm by 2019 across all hospital and clinic locations. A query of the electronic medical records of 8 hospitals was performed for the dates between January 1, 2018, and August 1, 2024, yielding a de-identified dataset. Inclusion criteria included all patients with the diagnosis of AIS who underwent PSF with instrumentation and had a valid prescription for an opioid. To the best of the authors' knowledge, all eight hospitals discharged patients with a prescription for an opioid. Encounters without a documented prescription were assumed to be incomplete records and excluded from query. Patients with complications such as surgical site infection, as determined by Current Procedural Terminology (CPT) or International Classification of Diseases (ICD) 10th Edition Procedure codes, present during admission were also excluded from query. We chose to exclude these patients as we were unsure of the effect that a prolonged hospitalization would have on discharge opioid prescriptions. 

Opioid prescriptions written from the day of admission to the day of discharge were considered the initial opioid prescription for a given surgery. If more than one prescription existed, the prescription closest to the time of discharge was chosen. Opioid prescriptions written between at-home days 1 and 30 following discharge are referred to as a refill. If two refill prescriptions existed within a 24-hour period, the later prescription was chosen. Multiple refills separated by 24 hours were considered distinct refills. Patients with a prescription for an opioid prior to admission for surgery were excluded as prior opioid exposure may change prescriber practice for at-home opioid prescribing. The total number of doses was calculated by dividing the total quantity prescribed (pills, tablets, capsules, or milliliters) by the amount to be taken per dose. The number of prescribed days was calculated by dividing the total number of doses by the maximum number of allowed per day if used as directed (every four hours as needed = maximum 6 times per day). Maximum daily MME was calculated as the MME per dose multiplied by the maximum number of daily doses available if taken as directed. Total prescription MME was calculated as MME per dose multiplied by the number of doses prescribed. The MME conversion chart is provided in Table 1.

**Table 1:** Opioid conversion table. Equivalent oral morphine strength in milligrams for a given milligram of opioid in the left column. MME: Morphine milligram equivalents

Opioid (Dose in mg)	MME Conversion Factor
Oxycodone	1.5
Hydromorphone	5
Tramadol	0.2

Patient demographics, including height, weight, body mass index (BMI), sex, race/ethnicity, and length of stay, were collected. Prescription data including prescriber, name of opioid, dose, and number of prescribed doses were collected. We included prescriptions for muscle relaxants and gabapentin in the query.

Descriptive statistics were calculated for continuous variables. Differences between groups were assessed using the Kruskal-Wallis test, and the Cochran-Armitage test was used to evaluate linear trends across ordinal variables. Significance was set at p < 0.05. Data were analyzed using R version 4.5.1 (R Core Team, Posit Team). 

## Results

We identified 1075 patients with AIS who underwent primary PSF with instrumentation across eight sites located across the United States (US) and were prescribed an opioid during their hospital stay. Of these patients, 41 had a prior opioid prescription within our dataset and were excluded. Of the remaining 1034 patients, the mean age was 15.4 ± 2.07 years. Female patients made up 76% (787/1034) of the study cohort. Mean BMI was 22.3 ± 5.6. The average length of stay was 3.96 ± 1.46 days. White was the largest cohort at 62% (638/1034). This was followed by Hispanic or Latino at 15% (153/1034), Black or African-American at 6% (66/1075), and Asian 5% (49/1075). The remain 12% of the cohort identified as American Indian or Alaska Native, Native Hawaiian/Other Pacific Islander, Multiple, Other, Unknown, or Declined.

Oxycodone made up 94% (977/1034) of all opioid prescriptions. Hydromorphone and tramadol were also prescribed. Seventy-seven percent of initial prescriptions (801/1034) were less than 50 MMED; only 5% greater than or equal to 90 MMED. Fourteen percent of patients (145/1034) received at least one refill prescription for opioids in addition to their initial prescription. Oral morphine equivalent details for all opioid prescriptions are provided in Table 2. 

**Table 2:** Opioid prescription details for 1075 patients with the diagnosis of adolescent idiopathic scoliosis who underwent primary posterior spinal fusion with instrumentation patients from January 1, 2018, to August 1, 2024. MME: Morphine milligram equivalents

Measure	Mean ± STD
Number of Doses Initially Prescribed	28.3 ± 10.16
MME Per Dose	8.58 ± 2.72
Maximum Doses per Day	5.53 ± 0.98
Maximum MME Per Day	47.26 ± 16.60
Total Prescribed Days	5.25 ± 1.97
Total MME Prescribed	240.6 ± 110.5

**Figure 1:**
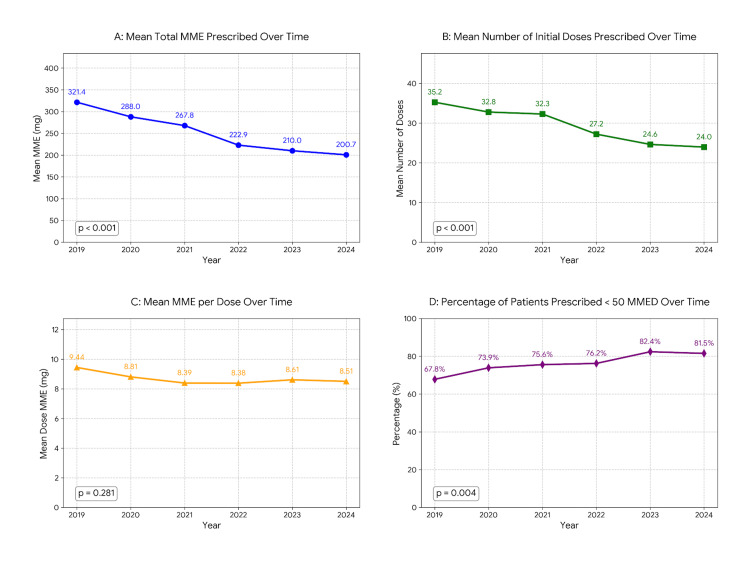
Opioid prescription details over time. A. Statistically significant decrease in the mean total MME prescribed (321.44 in 2019 to 200.73 in 2024, Kruskal–Wallis H = 119.33, p < 0.001). B. Statistically significant reduction in the mean number of doses initially prescribed (35.24 in 2019 to 23.95 in 2024, Kruskal–Wallis H = 145.94, p < 0.001). C. No statistically significant change in mean MME per dose (9.44 in 2019 to 8.51 in 2024, Kruskal–Wallis H = 6.27, p = 0.281). D. Statistically significant increase in the percentage of patients prescribed less than 50 MMED (67.8% in 2019 to 81.5% in 2024) increased over time (Cochran–Armitage trend test Z = 2.87, p = 0.004). MME: Morphine milligram equivalents; MMED: maximum morphine milligram equivalents daily

Over the course of our study period, there was a decrease in the mean total MME prescribed (321.44 in 2019 to 200.73 in 2024, Kruskal-Wallis H = 119.33, p < 0.001). This decrease was due to a reduction in the mean number of doses initially prescribed (35.24 in 2019 to 23.95 in 2024, Kruskal-Wallis H = 145.94, p < 0.001) as the mean MME per dose did not statistically change (9.44 in 2019 to 8.51 in 2024, Kruskal-Wallis H = 6.27, p = 0.281). The percentage of patients prescribed less than 50 MMED increased over this time (Cochran-Armitage trend test Z = 2.87, p = 0.004). Changes in opioid prescribing patterns over time are illustrated in Figure 1. 

As the distribution of case volume was not uniformly distributed across the sites for every year of the study (Table 3), we decided to only analyze the effect of time for each site on total MME prescribed and opioid refill rate. 

**Table 3:** Number of primary posterior spinal fusions with opioid prescription data done per site over time.

Year	Site 1	Site 2	Site 3	Site 4	Site 5	Site 6	Site 7	Site 8
2019	0	0	0	30	8	14	4	3
2020	1	5	3	43	42	35	13	15
2021	0	4	11	32	50	52	6	25
2022	6	2	21	30	60	43	43	43
2023	5	2	10	38	60	51	35	43
2024	0	15	5	17	41	23	17	28

Table 4 shows the mean total MME per initial prescription by site over time. There was statistically significant variation in the mean initial total MME prescribed at Sites 3, 4, 5, and 6 (Kruskal-Wallis test, H values in Table 4, p < 0.05). Site 5 showed a yearly decrease in mean total MME prescribed, while the other sites showed variation in the year-to-year changes. 

**Table 4:** Mean total MME per initial prescription by site over time. Statistically significant variation in the mean initial total MME prescribed over time at Sites 3, 4, 5, and 6 (Kruskal–Wallis test, H values reported in the table, significance level p < 0.05). MME: Morphine milligram equivalents

Year	Site 1	Site 2	Site 3	Site 4	Site 5	Site 6	Site 7	Site 8
2019	-	-	-	299.0	346.3	395.5	174.4	330.0
2020	280.0	187.5	162.5	256.3	321.6	334.9	146.5	357.0
2021		281.3	148.0	252.9	312.6	267.3	157.5	275.7
2022	436.7	187.5	114.9	186.5	270.7	168.0	209.1	275.1
2023	408.8	180.0	102.7	216.5	199.5	180.0	197.9	267.7
2024	-	269.5	91.5	221.9	180.5	145.1	178.5	259.3
p value	0.39	0.13	0.03	0.003	< 0.001	< 0.001	0.008	0.15
H value	1.89	7.12	4.83	17.87	84.79	120.65	15.61	8.07

Table 5 shows the rate of opioid refill by site over time. There was no statistically significant variation in the refill rate of opioids at each site over time (Cochran-Armitage test, Z value reported in Table 5, significance level p < 0.05). 

**Table 5:** Opioid refill rate by site over time. Statistically significant variation in the mean initial total MME prescribed between each site for every year of analysis (Cochran–Armitage test, Z value reported in the table, significance level p < 0.05).

Year	Site 1	Site 2	Site 3	Site 4	Site 5	Site 6	Site 7	Site 8
2019	-	-	-	13%	0%	0%	0%	33%
2020	0%	0%	0%	16%	0%	9%	23%	27%
2021	-	0%	27%	3%	6%	23%	17%	40%
2022	0%	50%	19%	13%	2%	37%	12%	14%
2023	40%	0%	0%	11%	3%	18%	14%	26%
2024	-	7%	20%	18%	5%	17%	24%	29%
p value	0.161	0.748	0.579	0.940	0.371	0.137	0.661	0.678
Z value	1.4	0.32	-0.56	-0.08	0.89	1.49	0.44	-0.42

## Discussion

In 2020, Harris et al. reviewed nationwide opioid prescribing practices following posterior spinal arthrodesis for adolescent idiopathic scoliosis from 2010 to 2016 [9]. They reported most opioid prescriptions complied with the 2016 CDC dosing guidance to avoid opioid prescriptions greater than 90 MMED [9]. During this study period, there was a measurable shift toward more conservative prescribing: the proportion of patients initially prescribed ≥ 90 MMED declined, and fewer patients filled multiple opioid prescriptions [9]. The primary finding of our study is that from 2018 to 2024, this trend has continued without a concomitant increase in the opioid refill rate. The decrease in the number of opioid doses initially prescribed was the primary driving factor in the statistically significant decrease in mean total MME prescribed over the study period. 

Harris et al. calculated MMED using the formula [9]: 

$\text{Strength per unit} * \left( \text{number of units/days supply}\right) * \text{MME conversion factor}$

In their 2020 study, 81% of patients who underwent posterior spinal arthrodesis for AIS in the US received prescriptions for opioids that complied with 2016 CDC guidelines [9]. While our prescription data did not have a “days supply” field available, we did have the frequency field (example: every six hours). Thus, we used a slightly different formula for calculating maximum daily MME: 

$\text{Strength per unit} * \text{Max number units available per day} * \text{MME conversion factor}$

Using this formula in our study population, 95% of patients were prescribed less than 90 MMED, and 77% were prescribed less than 50 MMED. Over the duration of our study, the percentage of patients prescribed less than 50 MMED statistically increased from 68% in 2019 to 82% in 2024. Additionally, Harris et al. reported an average of 78 +/- 71 opioid pills initially prescribed [9] while our dataset showed an average of 28.4 ± 10.21 doses of opioids prescribed. This is a clinically significant decline in the number of opioids prescribed from the study by Harris et al. (2010 - 2016) to our study (2019 - 2024). 

We choose to use CDC guidelines of 50 MMED and 90 MMED based on the precedent set by Harris et al. [9]. It should be noted that CDC guidelines regarding 50 MMED and 90 MMED were written for an adult population >= 18 years old. Opioid prescribing guidelines with specific numerical targets are currently lacking for the pediatric population. The lack of focused guidelines for adolescents undergoing specific surgical procedures highlights why further work is needed to establish evidence-based, age-specific, opioid prescribing guidelines.

More recent literature on opioid use following PSF for AIS suggests the number of opioid doses prescribed could be lowered further than our study average of 28 doses. Between December 2019 and July 2021, Garcia‑Munoz et al. set out to identify factors associated with increased opioid usage following discharge from PSF in AIS [10]. In their analysis of 27 patients, on average, 11.4 pills of oxycodone were used with an average of 45.4 doses prescribed [10]. This study did not identify any significant patient or surgical factors predisposing patients to increased home opioid use. From 2019 to 2021, Yang et al. studied the effect of preoperative patient education and standardization of 30 doses of oxycodone for postoperative pain in PSF for AIS patients. While the pre-intervention group used an average of 29 doses of oxycodone, the post-intervention group used fewer days (5.6 vs. 8.9, p < 0.001) and less oxycodone over the first week (14 vs. 23, p <0.001) [11]. This study showed that a smaller prescription quantity alone was associated with reduced consumption [11]. This idea is supported by the “portion size effect” theory as described by Marchiori et al. [12]. Here, patients adjust their own expectations on how many opioids they should be consuming based on the quantity prescribed. 

We found considerable variability in opioid prescribing practices and refill rates at our eight sites. During our study period, of the eight states involved in this study, one state had an 18-tablet (three days) limit on the number of opioids allowed in a prescription. One state had no limit on the number of opioids allowed, and six had a limit of three to seven days of opioids, with an exception for postsurgical patients. The effects of these different state laws on opioid prescribing practices, as well as patient education and expectations, require further study. 

A primary strength of this study is the use of a multi-institutional dataset collected over an extended period. The inclusion of eight sites, each operating with its own analgesic protocols, enhances the generalizability of our findings. However, the absence of documented site-specific surgical and postoperative analgesic pathways limits our ability to identify the specific drivers of the observed opioid prescribing variations.

The primary limitation of this study is our lack of data on patient opioid use following discharge for PSF. While we attempt to use the rate of opioid refills within the 30 days after surgery as a proxy for adequacy of pain control at home, our study would be stronger had there been patient-reported outcomes on pain and recovery available. Additionally, while we note the decreasing trend in the amount of opioids prescribed over time, we do not have a control population for which to compare our data set to. Furthermore, this study has limitations inherent in a retrospective study. Data quality and completeness can vary due to missing or inconsistent patient charting. There is also a risk of selection bias, as the study population depends on available records rather than randomized sampling. Finally, retrospective designs cannot establish causality, only associations, and are vulnerable to confounding factors that may not be adequately controlled. 

Our study adds to the work of Harris et al. by showing that prescribers, over time, continue to prescribe fewer opioids for PSF for AIS. Factors for declining opioid prescribing could include greater utilization of multi-modal non-opioid analgesia, an awareness by physicians and patients of the negative role that leftover opioids can play in the community, and changes in state prescribing laws. Further research is required to quantify the magnitude of the effect of these factors. Additionally, the lack of suggested opioid prescribing quantities for PSF, along with the variability in prescribing patterns seen across our eight sites, suggests that more work is needed to establish guidelines on optimal postoperative opioid usage. 

## Conclusions

Our multicenter analysis demonstrates a significant longitudinal decline in opioid prescribing quantities for adolescents undergoing PSF for AIS from 2019 to 2024. This trend was primarily driven by a reduction in the total number of doses dispensed rather than a decrease in the potency of individual doses. During the period, there was no statistically significant increase in opioid refills, suggesting that lower initial prescription quantities may be sufficient for postoperative pain management in this cohort without compromising clinical adequacy.

Persistent and statistically significant variability in prescribing patterns across our eight study sites highlights a lack of consensus on opioid prescribing and at-home management of post-operative pain. The development of standardized, procedure-specific prescribing protocols is needed to reduce the amount of unused opioids while ensuring optimized pain control.
